# Antioxidant and Hepatoprotective Effect of a Nutritional Supplement with Silymarin Phytosome, Choline Chloride, l-Cystine, Artichoke, and Vitamin E in Dogs

**DOI:** 10.3390/antiox11122339

**Published:** 2022-11-25

**Authors:** Claudia Giannetto, Francesca Arfuso, Elisabetta Giudice, Maria Rizzo, Giuseppe Piccione, Kamel Mhalhel, Maria Levanti

**Affiliations:** Department of Veterinary Sciences, Polo Universitario dell’Annunziata, University of Messina, 98168 Messina, Italy

**Keywords:** antioxidants, dogs, hepatoprotection, food supplement, silymarin phytosome

## Abstract

Phytotherapy has been promoted for the treatment of liver diseases in dogs. The interest in identifying the antioxidant/hepatoprotective potential of various plants is increasing. Every 10 days for 30 days, forty dogs were subjected to blood sampling and hepatic ultrasound assessment. Clinically healthy dogs (group A) and dogs with liver enzyme and ultrasound hepatic aspects and sizes outside the physiological range (group B) were divided into two subgroups. Every day for 30 days, one subgroup received Epacare pet + pasta^®^ and the other received a placebo. Differences due to groups were observed in dROM, SHp, AST and LDH serum concentrations. The administration of Epacarepet + pasta^®^ for 30 days had an influence on the serum concentration of dROMs, SHp, AST, ALT, ALP, LDH, and urea. The application of paired Student’s t-test showed a decrease in the longitudinal and transverse liver axis size. In conclusion, feed supplementation with Epacare pet + pasta^®^ had a beneficial effect on the antioxidant status and liver enzymes in animals with liver enzymatic alterations and in healthy dogs.

## 1. Introduction

The liver is a central organ in the regulation of the metabolism and detoxification of noxious agents. It also plays a central role in the regulation of hepatocyte regeneration and systemic inflammatory responses. Acute and chronic liver diseases due to the exposure to a deleterious external stimulus that exceeds the protective and repair capacity of the liver are accompanied by some degree of inflammation [1]. Hepatocytes and non-parenchymal liver cells activate intrinsic defense mechanisms of enzymatic and non-enzymatic detoxification aimed at neutralizing insult or damage, as a response to exposure to drugs, chemicals and xenobiotics [2]. In the presence of liver diseases, hepatocyte mitochondria and the endoplasmic reticulum are sites of reactive oxygen species (ROS) production. It is well known that ROS production is counterbalanced by the “antioxidant systems” responsible for the protection of cells from the actions of free radicals. In cases of excessive ROS production, this system fails and cell injuries occur [3].

In recent decades, in human medicine, phytotherapy has received increasing attention; the treatment of liver diseases with compounds of plant origin seems to be one of the most attentive aspects on the basis that phytotherapy is safe because they are “natural” and fit into the image of a gentle and, therefore, a harmless alternative to conventional medicine [4].

Since the 16th century, silybin has been used to treat liver diseases and it has been proven to be effective in acute and chronic conditions in humans, dogs, and cats [5]. Silybin is a flavonoid that together with silibinin, silidianin, silichristin, and isosilibinin constitute Silybum marianum (Milk thistle) [6]. The pharmacological profile of silymarin has been well defined. At a dosage ranging from 10 µM to 300 µM, silymarin had antioxidant, hepatoprotective, anti-inflammatory, and anti-fibrotic effects [5]. The hepatoprotective properties of silymarin were investigated both in vitro and in vivo. Experimental studies demonstrated its antioxidant and free radical scavenging properties, its ability to improve antioxidative defense by prevention of glutathione depletion, and its antifibrotic activity [4]. In dogs, liver diseases have a high prevalence; in these animals, proper nutritional care should be given, as in dogs affected by heart, renal or gastrointestinal disorders that receive special diets [7].

Epacare pet + pasta^®^ (Gefarma, Acireale, Italy) is a food supplement based on choline chloride, vitamin E, l-cistine, milk thistle (Silymarin Phytosome^®^, Gefarma Italia, Acireale, Italy) and artichoke extracts. It is used in cases where it is necessary to improve liver function, such as acute and chronic hepatitis, hepatic insufficiency and intoxication. Within its components, Silymarin Phytosome^®^ is considered the active component; it is a liposomal complex, which allows greater bioavailability of silymarin, in order to improve clinical efficacy in liver protection. The Phytosome^®^ administration system has also been applied to silymarin with promising preclinical results that are consistent with the oral bioavailability of most of the flavanolignans present in the extract. These data enable us to optimize the dose for clinical efficacy in liver protection and pave the way for the use of silymarin in new therapeutic areas, where recent findings indicate an important role for the entire phytocomplex. Choline chloride, vitamin E, l-cistine, and artichoke extracts are bioequivalent components added as additives that not only have hepatoprotective effects, but also act in other ways.

However, few studies have been conducted on the supplementary feed that supports liver function in dogs. The wide presence of commercial food integrated with silymarin is not supported by scientific reports on this topic. On the basis of this, the aim of the present study was to investigate the liver response to diet supplementation with a type of nutraceutical food based on choline chloride, l-cystine, silymarin phytosome, artichoke, and vitamin E in dogs.

## 2. Materials and Methods

### 2.1. Animals and Sampling Protocol

Forty mixed-breed dogs (21 neutered males; 19 neutered females) with a mean body weight of 28 ± 5 kg, aged between 2 and 5 years old, living in multiple dog boxes in a shelter, were enrolled in the study. All dogs received the same commercial food (cereals, meat and animal by-products (of which 4% included 5 types of meat), oils and fats (of which 0.2% was fish oil and 0.2% sunflower oil), vegetable by-products (of which 2% was beet pulp), minerals (of which 0.7% was pentasodium triphosphate); in addition to crude protein (22.0%), crude fat (13.0%), crude fiber (2.5%), crude ash (7.5%), calcium (1.4%) and phosphorus (1.0%) supplemented with vitamin A (17,350 IU/kg), vitamin B1 (8.2 mg/kg), vitamin B2 (14.8 mg/kg), vitamin (B3 34.7 mg/kg), vitamin B5 (33.2 mg/kg), vitamin (B6 3.8 mg/kg), vitamin B9 (0.77 mg/kg), vitamin B12 (0.07 mg/kg), vitamin D3 (1065 IU/kg), vitamin E (280 mg/kg), biotin (0.33 mg/kg), anhydrous calcium iodate (2 mg/kg), copper sulphate pentahydrate (55 mg/kg), manganese II sulphate monohydrate (220 mg/kg), sodium selenite (0.45 mg/kg) and zinc sulphate monohydrate (560 mg/kg)) used in the shelter as the usual food, and this supplied to each animal once a day (12.00). Water was available ad libitum. The day before the start of the study, 70 dogs living in the shelter were subjected to clinical examination, laboratory tests, including complete hematological and biochemical profiles, and hepatic ultrasound assessment (Esaote MyLabFivevet, Linear transducer 7.5–12.0 MHz). On the basis of the pre-enrollment check, the animals were divided into 2 equal groups. Group A comprised 20 clinically healthy dogs (10 neutered males and 10 neutered females), with hematological and hematochemical parameters within the physiological range for dogs [8], and ultra-sound hepatic aspects and sizes within the physiological range for the canine species [9]. Group B (hill dogs) comprised 20 dogs (11 neutered males and 9 neutered females) with liver enzyme (aspartate amino transferase (AST), alanine aminotransferase (ALT) and alkaline phosphatase (ALP)) levels outside the physiological ranges indicated for the canine species and ultrasound hepatic aspects and sizes outside the physiological range for the canine species. Each group was divided into two sub-groups of ten subjects. One subgroup (A_T_ and B_T_) received every day for 30 days 1 g/10 kg of their body weight of Epacare pet + pasta^®^ (Gefarma, Italy), which was added to the usual food used in the shelter. The other subgroups (A_C_ and B_C_) received every day for 30 days 6 cm of a placebo (oil–fish mixture), which was added to the usual food used in the shelter.

This study received the Department’s Animal Ethics Council approval (protocol number: 61-2021). All treatments, housing, and animal care reported in this study were carried out in accordance with the EU Directive 2010/63/EU for the protection of animals used for scientific purposes.

### 2.2. Sample Collections

Every 10 days for 30 days (T0-T1-T2-T3), each dog from every group was subjected to blood sampling at the same hour of day (9.00 a.m.). At T0 and T3, liver ultrasound with a 5 MHz probe was performed on each dog, in lateral recumbency. The pre-enrollment check was considered as T0. Blood samples were taken by the shelter technical staff. The samples were collected by brachial venipuncture using two vacutainer tubes. One of these tubes with EDTA anticoagulant was used for hematological assessment; the other tube with a clot activator was used for the assessment of serum parameters. For the sera obtainment, tubes were left at room temperature for at least 30 min and then centrifuged at 3000 rpm for 7 min. The obtained sera were stored at −20 °C before analysis. By visual inspection, all the obtained sera were confirmed as non-hemolyzed. Serum levels of dROMs, SHp, AST, ALT, ALP, lactate dehydrogenase (LDH), total bilirubin, creatinine, urea, glucose, and total protein were determined using a UV spectrophotometer (SEAC, Slim, Florence, Italy). The values of dROMs and SHp were assessed with the so-called “spin traps” system (Diacron International, Milan, Italy) and the other parameters were assessed with the use of commercial kits (Byosistems, Reagents and Instruments, Barcelona, Spain). In addition, at T0, creatine kinase (CK) was assessed by means of a UV spectrophotometer (Slim SEAC, Florence, Italy) in order to exclude some muscular damage that contributed to the AST increase. The liver ultrasound was performed by a vet clinician expert in this field that was blinded to the patient’s group allocation.

### 2.3. Statistical Analysis

The data obtained were normally distributed (Kolmogorov–Smirnov test). To compare the data obtained for each tested parameter at various data points (T0-T1-T2-T3) and between the different experimental groups (A_T_-A_C_-B_T_-B_C_), two-way repeated measures analysis of variance (ANOVA, GraphPad Prism 9.0) and the Bonferroni post-hoc comparison test were performed. Paired Student’s *t* test was applied to compare the liver size at T0 and T30 *p* values < 0.05 were considered statistically significant. The results are expressed as mean ± standard deviation (SD). Statistical analysis was performed by using the calculation software Prism 9.0 (Graph Pad Software, San Diego, CA, USA).

## 3. Results

Blood screening performed during the pre-enrollment check is reported in Table 1. Hematological parameters (red blood count—RBC, hematocrit—Hct, hemoglobin—Hgb, platelet—PLT, and white blood count—WBC); and serum creatinine, urea, glucose, total protein, and albumin were within the physiological range reported for dogs. Serum LDH and total bilirubin were lower than the physiological range reported for dogs in some animals of group B (hill dogs).

**Figure 1:**
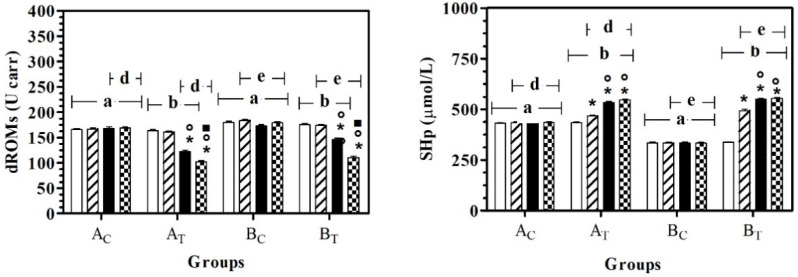
Mean ± standard deviation (SD) of dROMs and SHp recorded for the different data points (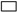 T0; 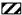 T1; 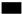 T2; 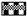 T3) of the experimental protocol, in all groups (Ac = control not treated; A_T_ = control treated; B_C_ = hill not treated and B_T_ = hill treated). Symbols indicate statistical differences due to time, within the same group (* vs. T0; ° vs. T1; ▪ vs. T2). The same lowercase letter indicates statistical differences due to treatment between groups.

The application of two-way ANOVA showed a significant effect of the group on dROMs (F_(3,108)_ = 119.20; *p* < 0.0001), SHp (F_(3,108)_ = 960.80; *p* < 0.0001), AST (F_(3,108)_ = 46.43; *p* < 0.0001) and LDH (F_(3,108)_ = 13.60; *p* < 0.0001), and a significant effect of treatment (time) on dROMs (F_(3,108)_ = 194.70; *p* < 0.0001), SHp (F_(3,108)_ = 340.30; *p* < 0.0001), AST (F_(3,108)_ = 40.49; *p* < 0.0001), ALT (F_(3,108)_ = 36.12; *p* < 0.0001), ALP (F_(3,108)_ = 17.57; *p* < 0.0001), LDH (F_(3,108)_ = 2.37; *p* < 0.05) and urea (F_(3,108)_ = 6.27; *p* = 0.0006). No statistically significant modifications were observed for total protein or albumin. In particular, dROMs and SHp were statistically higher in A_C_ and B_C_ compared to A_T_ and B_T,_ respectively. In both groups, a significant effect of time on dRoms was observed starting from T2, with respect to T0 and T1; a significant effect of time on SHp was observed starting from T1, with respect to all the previous experimental conditions (Figure 1).

**Figure 2:**
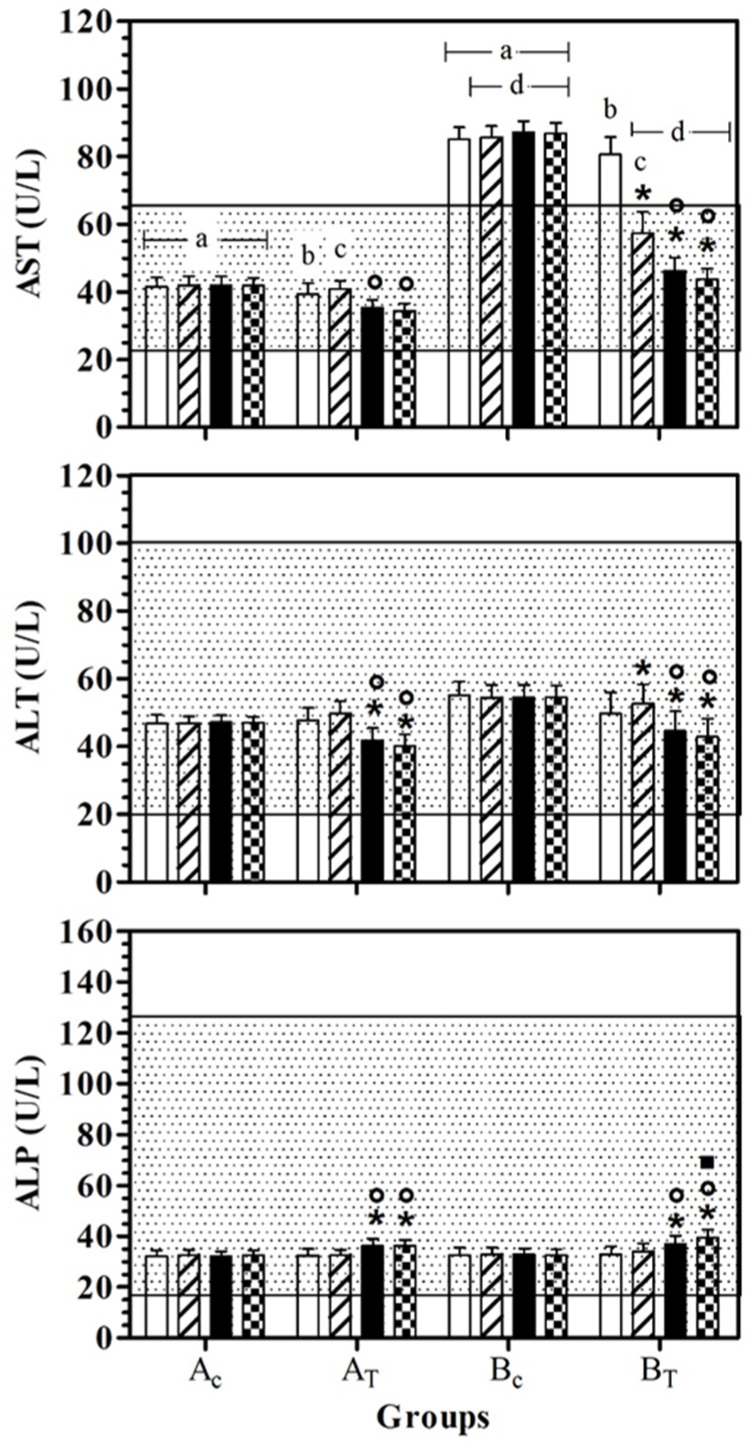
Mean ± standard deviation (SD) of serum liver enzymes used as inclusion criteria (aspartate aminotransferase—AST, alanine aminotransferase—ALT; alkaline phosphatase—ALP) recorded for the different data points (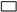 T0; 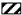 T1; 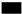 T2; 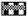 T3) of the experimental protocol, in all groups (Ac = control not treated; AT = control treated; BC = hill not treated and BT = hill treated). Dotted area indicates the dog physiological range. Symbols indicate statistical differences due to time, within the same group (* vs. T0; ° vs. T1; ▪ vs. T2). Same lowercase letter indicates statistical differences due to treatment between groups.

AST values were statistically lower in A_C_ than B_C_, at all data points. In B_T_, AST serum values were lower than B_C_, at T1, T2, and T3. At T0 and T1, the AST serum value was statistically higher than for A_T_. A significant effect of treatment was observed in healthy dogs (A_T_) at T2 and T3 and the AST serum values were statistically lower than for T1. In hill dogs (B_T_), AST serum values were statistically lower at T1, T2, and T3 than T0, and at T2 and T3 compared to T1. ALT serum values were statistically lower at T2 and T3 than T0 and at T1 in A_T_ and B_T_. In B_T_, the ALT serum value was statistically higher at T1 than at T0. ALP serum values were statistically higher at T2 and T3 than T0 and at T1 in AT and BT. In B_T_, the ALP serum value was statistically higher at T3 than T2. Figure 2 shows the mean ± standard deviation (SD) of the serum liver enzymes used as the inclusion criteria (AST, ALT and ALP) recorded for the different data points of the experimental protocol, in all groups.

**Figure 3:**
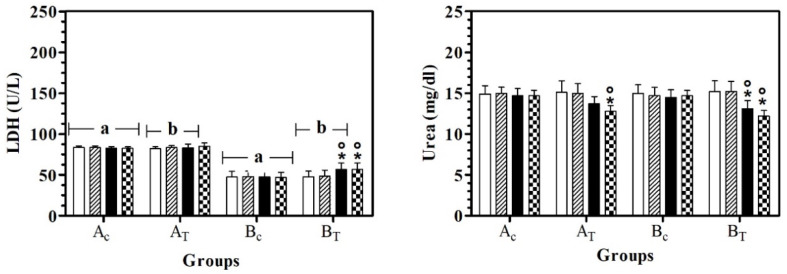
Mean ± standard deviation (SD) of serum lactate dehydrogenase (LDH) and urea recorded for the different data points (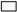 T0; 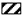 T1; 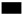 T2; 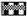 T3) of the experimental protocol, in all groups (Ac = control not treated; A_T_ = control treated; B_C_ = hill not treated and B_T_ = hill treated). Symbols indicate statistical differences due to time, within the same group (* vs. T0; ° vs. T1). Same lowercase letter indicates statistical differences due to treatment between groups.

LDH values were statistically higher in A_C_ than B_C_, at all data points. In B_T_, LDH serum values were lower than A_T_, at T0, T1, and T2. A significant effect of treatment was observed in hill dogs (B_T_) and LDH serum values were statistically higher at T2 and T3 than at T0 and T1. Urea serum values were statistically lower at T3 than T0 in A_T_ and B_T_ and in B_T,_ the urea serum value at T2 was lower than T0 and T1 (Figure 3).

**Figure 4:**
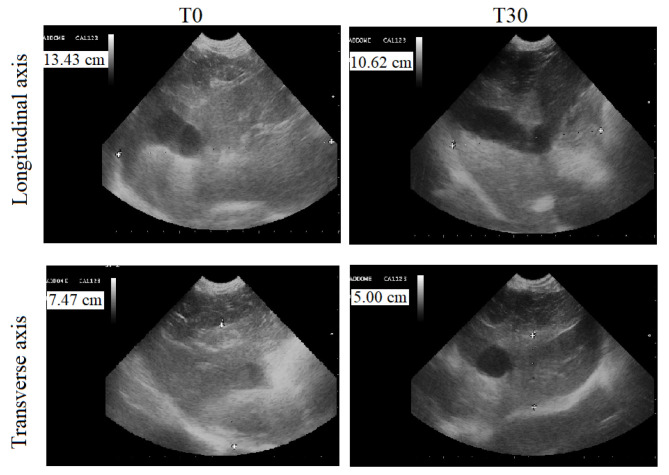
Liver ultrasound performed by a 5 MhZ probe, at T0 (before the beginning of treatment) and T3 (30 days after the administration of Epacare pet + pasta^®^) of a representative dog from the B_T_ group.

Abdominal ultrasounds of the dogs’ livers showed a homogeneous parenchymal pattern and echoic tissue, in addition to normal hepatic ducts and biliary tracts in group Ac and A_T_. The longitudinal axis (dorso-ventral) was between 9.00 and 8.00 cm, and the transverse axis (cranio-caudal) was between 6.50 and 5.50 cm. No changes were observed during the experimental protocol period. At T0, the ultrasonographic features of the livers of group B animals included hyperechoic parenchymal patterns and the presence of small fibrotic hyperechoic spots. The hepatic duct and biliary tract of these animals were slightly enlarged. The ultrasonographic features of the livers of the animals of the treated group (B_T_) observed at T0 and at T3 are reported in Table 2. The application of the paired Student’s *t*-test showed a statistically significant decrease in longitudinal (*p* < 0.0001) and transverse (*p* < 0.0001) axis size. Figure 4 displays a representative ultrasonographic image of the longitudinal and transverse axis of a dog of the B_T_ group at T0 and T3.

## 4. Discussion

Phytotherapeutic nutritional supplements can be used as an alternative and/or complementary medicine, for example, to prevent the development of a disease.

The most important antioxidant mechanism of sylimarin is its ability to inhibit the enzymes involved in the production of ROS, preventing free radical formation [12]. At T0, the pattern of oxidant/antioxidant activity was characterized by high dROM values and low SHp values outside the physiological range reported for dogs, as previously reported for dogs living in shelters [13]. Shelters are not able to accommodate every unwanted dog and a reduction in motor and relationship activity increases the state of anxiety of the animals, leading to increased values of dROMs that cannot be solved by the production of antioxidants [14].

Following t administration of sylimarin for 30 days, values within the physiological range were reported, with the observed effect starting 10 days after the food supplementation. Silymarin contributes to the antioxidant effect through direct free radical scavenging, by preventing free radical production and by activating a range of antioxidant enzymes [15].

Silymarin’s therapeutic potential is based on its ability to reduce liver enzyme activity. Phytotherapeutic nutritional supplements can be very effective both for preventive purposes and as support for the treatment of some pathologies. The investigation of a group of healthy dogs allowed us to investigate the use of silymarin for preventive purposes; the investigation of a group with mild liver alterations also allowed us to investigate the use of silymarin as treatment or support for the treatment of liver pathologies.

As previously observed by Gogulski et al. [16], feed supplementation with silymarin positively affected liver conditions. The serum levels of the liver enzymes used as part of the inclusion criteria statistically changed in both treated groups (A_T_, B_T_). AST, a cytoplasmatic and mitochondrial enzyme found in the liver, brain, myocardial, and skeletal muscle cells, decreased following the first 10 days after the Epacare pet + pasta^®^ supplementation and reached serum levels similar to those for A_C_ at 30 days after the start of feed supplementation. Its elevation in serum is linked to the cellular damage that led AST to diffuse into the extracellular compound [17]. Its concentration was higher than the physiological range only in the B groups (B_C_ and B_T_). In addition, in the A_T_ group, a statistical decrease after 20 and 30 days of feed supplementation was recorded. As is the case with AST, ALT is a cytoplasmatic enzyme that is widely used as a hepatic biomarker of health and is present not only in hepatocytes, but also in skeletal and cardiac muscle [17]. Its concentration was within the physiological range in all groups during the experimental period. As previously reported in dogs clinically affected by liver damage [16], silymarin supplementation induced a statistical decrease in ALT serum levels, starting from 20 days after the feed supplementation and persisting until the end of the study. ALP, the index of cholestasis in dogs, in which serum levels increase before the total bilirubin serum levels increase [16], was reported to be within the physiological range for the entire experimental period and statistically increased after 20 days of silymarin supplementation, remaining higher than the value reported at T0 and T1 until the end of the study.

LDH serum concentration was statistically lower in the B group than A group. LDH is an enzyme that catalyzes the reversible conversion of L-lactate to pyruvate. It is present in all tissues. Its activity is not specific; however, the liver, muscles, and erythrocytes are sources of high activity. Its serum values were lower in the B groups than A groups. The lower value in dogs with hepatic diseases could be attributed to the reduced glycolytic activity of hepatic cells, due to a reduction in blood flow in the first stage of hepatic problems [18]. In the B_T_ group, LDH increased 20 days after feeding supplementation, underlining the ability of silymarin to re-establish the physiological conditions in the liver. Our results disagree with a previous study [19] that reported the absence of an influence of sylimarin administration on the values of LDH, BUN and the urea ratio.

Urea serum levels decreased in both treated groups at 30 days after feed supplementation. In particular, in the B_T_ group, the urea decrease started 20 days after feed supplementation. Our results were in accordance with those reported by Goguloski et al. [16], who observed a decrease in urea serum concentration 30 days after the administration of an herbal preparation with silybin used as a bioactive compound, at a dose of 28.3 mg/10 kg of body weight. The reason why a decrease in serum urea concentration can be used as an index of better liver functionality has not been elucidated.

The exact determination of liver size is required in clinical practice, since changes in liver volume can provide information about changes in liver parenchyma and could, therefore, play an important role in therapeutic decisions. In addition, 30 days of oral supplementation of silymarin led to a statistically significant reduction in the longitudinal and transverse liver axis size, which was associated with a reduction or disappearance of hyperechoic spots. All the animals that received silymarin supplementation tolerated the compound well; no gastrointestinal side effects due to silymarin administration [20] were observed.

## 5. Conclusions

In conclusion, we can conclude that feed supplementation with Epacare pet + pasta^®^ had a beneficial effect on liver function. The positive effect on the liver was observed not only in animals with mild liver enzymatic alterations, but also in healthy dogs. This confirms the safe use of feed supplementation in dogs, which can be used as supportive therapy for mild liver diseases, and as preventive support in dogs exposed to potential noxae.

## Figures and Tables

**Table 1:** Blood screening performed during the pre-enrollment check. Hematological parameters (red blood count—RBC, hematocrit—Hct, hemoglobin—Hgb, platelet—PLT and white blood count—WBC); reactive oxygen species (dROMs), thiol antioxidant barrier (SHp), serum liver enzymes used as inclusion criteria (aspartate aminotransferase—AST, alanine aminotransferase—ALT; alkaline phosphatase—ALP) are shown using grey bars, and serum lactate dehydrogenase (LDH), total bilirubin, creatinine, urea, glucose, total protein and albumin are expressed in their conventional unit.

		Experimental Group			
		Group A		Group B	
Parameters	Normal Range [8,10,11]	Control	Treatment	Control	Treatment
Reb blood count (RBC)—×10^6^ µL	5.50–8.50	7.46 ± 0.64	7.39 ± 0.83	7.55 ± 0.70	7.25 ± 0.56
Hematocrit (Hct)—%	37.00–55.00	53.86 ± 7.02	53.63 ± 7.04	53.58 ± 5.68	53.60 ± 4.17
Hemoglobin (Hgb)—g/dL	12.00–18.00	16.24 ± 1.04	16.08 ± 1.59	16.50 ± 1.20	16.64 ± 0.86
Platelet (PLT)—×10^3^ µL	200.00–500.00	301.78 ± 101.25	298.15 ± 108.38	305.25 ± 105.15	316.28 ± 102.35
White blood count (WBC)—×10^3^ µL	6.00–17.00	8.45 ± 1.16	8.68 ± 1.45	8.48 ± 1.35	8.31 ± 0.96
Reactive oxygen species (dROMs)—(U carr)	56–91	165.39 ± 6.69	163.79 ± 5.62	179.20 ± 9.51	175.10 ± 7.85
Thiol antioxidant barrier (SHp)—µmol/L	450–650	430.54 ± 10.19	433.60 ± 12.78	335.10 ± 11.51	337.16 ± 9.58
Aspartate aminotransferase (AST)—U/L	23.00–66.00	41.50 ± 8.79	39.40 ± 10.15	85.10 ± 11.23	80.50 ± 16.53
Alanine aminotransferase (ALT)—U/L	21.00–102.00	46.80 ± 8.05	47.70 ± 11.57	55.10 ± 12.88	49.60 ± 20.15
Alkaline phosphatase (ALP)—U/L	20.00–156.00	32.10 ± 7.63	32.40 ± 8.50	32.60 ± 9.33	32.90 ± 10.31
Lactate dehydrogenase (LDH)—U/L	45.00–233.00	83.40 ± 6.39	82.20 ± 7.56	47.40 ± 21.94	47.50 ± 22.75
Creatinkinase (CK)—U/L	1.15–28.40	15.21 ± 2.38	13.58 ± 3.75	16.39 ± 3.05	14.78 ± 2.45
Total Bilirubin—mg/dL	0.06–0.12	0.09 ± 0.17	0.09 ± 0.007	0.09 ± 0.09	0.10 ± 0.07
Creatinine—mg/dL	0.50–1.50	0.92 ± 0.26	0.92 ± 0.26	0.86 ± 0.23	1.00 ± 0.38
Urea—mg/dL	10.00–20.00	14.90 ± 3.17	15.10 ± 4.45	15.00 ± 3.23	15.20 ± 4.15
Glucose—mg/dL	65.00–118.00	94.40 ± 15.88	88.30 ± 22.90	75.60 ± 6.83	76.70 ± 9.14
Total Protein—g/dL	5.40–7.10	6.25 ± 0.54	6.51 ± 0.36	6.52 ± 0.60	6.45 ± 0.49
Albumin—g/dL	2.60–3.30	2.94 ± 0.24	2.85 ± 0.27	2.92 ± 0.23	2.83 ± 0.43

**Table 2:** Ultrasound features (parenchymal pattern, hepatic duct aspect, biliary tract aspect and liver size recorded on the longitudinal and transverse axis, expressed in cm) observed in each animal of the treated hill dog (B_T_) group, at T0 (before the beginning of treatment) and T3 (30 days after the administration of Epacare pet + pasta^®^).

B_T_	Ultrasound Features									
	T0					T3				
Animal	Parenchymal Pattern	Hepatic Duct	Biliary Tract	Longitudinal Axis (cm)	Transverse Axis (cm)	Parenchymal Pattern	Hepatic Duct	Biliary Tract	Longitudinal Axis (cm)	Transverse Axis (cm)
1	Hyperechoic, small fibrotic hyperechoic spots	Enlarged	Normal	13.45	7.50	Slightly hyperechoic, small fibrotic hyperechoic spots	Normal	Normal	10.62	5.00
2	Hyperechoic	Normal	Enlarged	15.30	10.67	Slightly hyperechoic	Normal	Normal	13.40	8.50
3	Hyperechoic	Enlarged	Normal	12.70	11.86	Normal	Normal	Normal	8.94	7.61
4	Hyperechoic	Normal	Enlarged	11.05	10.08	Normal	Normal	Normal	8.70	6.52
5	Hyperechoic, small fibrotic hyperechoic spots	Normal	Normal	12.07	9.85	Normal, small fibrotic hyperechoic spots	Normal	Normal	8.92	7.45
6	Hyperechoic	Enlarged	Normal	13.45	10.42	Slightly hyperechoic	Normal	Normal	9.50	7.77
7	Hyperechoic	Normal	Enlarged	12.98	9.85	Normal	Normal	Normal	9.70	7.23
8	Hyperechoic	Normal	Normal	15.45	10.12	Normal	Normal	Normal	10.45	8.20
9	Hyperechoic, small fibrotic hyperechoic spots	Enlarged	Normal	15.78	10.96	Slightly hyperechoic, small fibrotic hyperechoic spots	Normal	Normal	10.23	8.12
10	Slightly hyperechoic	Enlarged	Normal	12.36	9.87	Normal	Normal	Normal	9.48	7.10
11	Slightly hyperechoic	Normal	Enlarged	12.02	8.56	Normal	Normal	Normal	8.79	6.52
12	Slightly hyperechoic, small fibrotic hyperechoic spots	Normal	Normal	15.78	7.89	Normal, small fibrotic hyperechoic spots	Normal	Normal	10.32	5.58
13	Slightly hyperechoic	Enlarged	Normal	14.99	8.98	Normal	Normal	Normal	11.02	6.45
14	Slightly hyperechoic	Normal	Enlarged	12.63	7.85	Normal	Normal	Normal	9.89	5.15
15	Slightly hyperechoic	Normal	Enlarged	11.98	8.00	Normal, small fibrotic hyperechoic spots	Normal	Normal	8.78	5.28
16	Slightly hyperechoic	Enlarged	Normal	12.45	9.56	Normal	Normal	Normal	9.45	6.87
17	Slightly hyperechoic, small fibrotic hyperechoic spots	Normal	Enlarged	12.63	9.12	Normal, small fibrotic hyperechoic spots	Normal	Normal	9.23	6.23
18	Hyperechoic	Enlarged	Normal	11.28	7.58	Slightly hyperechoic	Normal	Normal	8.69	5.10
19	Hyperechoic	Normal	Enlarged	11.75	7.99	Normal	Normal	Normal	8.54	5.19
20	Hyperechoic, small fibrotic hyperechoic spots	Enlarged	Normal	16.03	10.98	Small fibrotic hyperechoic spots	Normal	Normal	11.87	7.35

## Data Availability

The data presented in this study are available on request from the corresponding author.
